# Cataract surgery with intraocular lens implantation in children aged 5-15 in local anaesthesia: visual outcomes and complications

**DOI:** 10.11604/pamj.2016.24.200.9771

**Published:** 2016-07-07

**Authors:** Kagmeni Giles, Domngang Christelle, Bilong Yannick, Otto Herrmann Fricke, Peter Wiedemann

**Affiliations:** 1 University Teaching Hospital Yaoundé (UTHY), Cameroon; 2 University of Yaoundé I, Faculty of Medicine and Biomedical Sciences, Cameroon; 3 Mountains University Banganté, Cameroon; 4 Department of Ophthalmology and Department of Clinical and Experimental Medicine, Linkoping University, Sweden; 5 Eye Hospital of Leipzig University, Germany

**Keywords:** Pediatric cataract, local anesthesia, intraocular lenses, postoperative complications

## Abstract

The aim of this study was to report feasibility, the visual outcomes and complications of pediatric cataract surgery with primary intraocular lens implantation in children aged 5 to15 years in local anesthesia. This retrospective interventional case series included 62 eyes from 50 children who underwent pediatrc cataract surgery with primary intraocular lens implantation at the Mana eye Clinic Nkongsamba between 2006 and 2015 Main outcome measures were: best-corrected post operative visual acuity, and intraoperative and postoperative complications. Mean age at surgery was 10.18 ± 3.21 years. Mean follow up length was 15.75 ± 3.36 weeks. Etiology included: 10 congenital cataracs (16.12%). 35 developmental cataracts (56.45%) and 17 traumatic cataracts (27.41%). The mean preoperative BCVA was logMAR 1.19 ± 0.33. (range 0.6-2.3). After cycloplegia refraction 2 weeks after surgery, the mean postoperative BCVA was log MAR 0.58 ± 0.88 ( range 0.5-1.8). The mean implanted IOL power was 22.01 ±3.16 D. IOL was succefuly implanted in 54 eyes (87.07%). Eight eyes (9.67%) were left aphakic. Increase in BCVA of 4 logMAR lines and above was recorded in 27 patients (43.55%). Intraoperative complications included: 4 posterior capsule holes with vitrous lost, 3 lenses subluxation and 1 case of iris dialyse. Late postoperative complications included: posterior capsular opacity which occurred in 16 patients, 3 posterior synechia, 2 retinal detachment. Peribulbar anaesthesia can be considered as a viable option in selected patients presenting developmental cataract undergoing cataract surgery in developing countries. Effort should be made to improve the early identification of congenital cataract and its early surgical intervention and prompt optical rehabilitation to prevent amblyopia.

## Introduction

Cataract is a common cause of childhood blindness [1, 2]. The prevalence of childhood blindness is approximately 10 times higher in sub-Saharan Africa than in developed countries [1]. The following two types of childhood cataract have been described: A congenital cataract presents either from birth or shortly thereafter. A developmental cataract usually refers to a cataract that appears age of 2 years [2]. Despite the rapid development in recent years of techniques for paediatric cataract surgery, the management of paediatric cataracts remains challenging in sub-Saharan Africa because of inadequate infrastructures and supplies and a lack of general anaesthesia facilities. The visual outcome after surgery depends on many factors, including ocular characteristics, early diagnosis and intervention and adequate visual rehabilitation. Encouraged by the results reported by Fan DS et al. in China [3], we started to use local anaesthesia in paediatric cataract treatment. The purpose of the present study is to describe the visual outcomes and complications of cataract surgery with primary intraocular lens (IOL) implantation in children aged 5 to 15 years under local anaesthesia.

## Methods

The medical charts and operation protocols of all children diagnosed with cataract who underwent cataract surgery with IOL implantation at the Mana Eye Clinic Nkongsamba between 2007 and 2015 were retrospectively reviewed. This research was approved by the local ethics committee. (Protocol 020/16). Prior to surgery, written informed consent was obtained from all parents or legal relatives after receiving an explanation of the nature of the procedure. Demographic parameters (i.e., gender, age and residency) and clinical characteristics (i.e., type of cataract, pre- and post-best corrected visual acuity (BCVA), intraocular pressure (IOP), type of surgery, power of the implanted IOL, pre- and postoperative complications and compliance for follow-up visits) were analysed. The BCVA was tested on a Snellen chart and was converted to the logarithm of the minimum angle of resolution (logMAR) for statistical purposes. Non-numerical vision was arbitrary assigned a logMAR value So, counting finger (CF) = logMAR 1.70, hand motion (HM) = logMAR 2.00 intact light perception = logMAR 2.30, defect light perception = logMAR 2.70, and no light perception (NLP) = logMAR 3.00. The IOP was measured with an eye care tonometer. Strabismus was checked using alternate cover testing. Biomicroscopy was carried out before dilatation. A dilated fundus examination was done with a direct or indirect ophthalmoscope. Cataracts that impeded the vision of the red reflex at the ocular fundus examination were considered as total. These patients underwent a B-scan sonography to assess the retinal status. A patient was classified as having a congenital cataract if a cataract was noticed before the age of 1 year or a presence of strabismus or nystagmus was observed. All other non traumatic cases were considered as developmental cataract.

Exclusion criteria included active uveitis, 360-degree posterior synechia, glaucoma, mental retardation, non cooperation with local anaesthesia and no concrete light projection. The IOL power was calculated using the Holliday II formula based on axial length (A-Scan) and keratometry readings. Depending on the age of the child, a specific amount of undercorrection to determine the power of the implanted IOL was done according to Dahan et al [4] who recommend an undercorrection of 20% for patients less than 2 years old and 10% for patients from 2 to 8 years old. Since all participants were 5 years old and above, only an undercorrection of 10% was applied in this series. When keratometry was not available, a standard 21D lens was implanted.

### Procedure

Positive psychological preparation of the patient was mandatory. The presence of the parents, from the consulting room to the operating room, was required. In our eye clinic, the following two standard operation techniques are used for paediatric cataract surgery under local anaesthesia according to the availability of IOLs: small incision cataract surgery (SICS) and manual phaco aspiration (MPA). The SICS was performed as described in the literature [5]. The MPA was performed in the following miner. Local anaesthesia consisted in a retrobulbar or peribulbar injection, with 4 ml of 50% of lidocaine 2% and a bupivacaine mixture. After disinfecting the skin and conjunctiva, two 2.8- to 3.0-mm clear corneal tunnel incisions were made at the 9 o’clock and 12 o’clock positions with a keratoma. Adrenaline (1:500,000) was usually added in the BSS to maintain the pupillary mydriasis. A viscoelastic substance was used to reform the anterior chamber. A 4- to 5-mm diameter anterior continuous curvilinear capsulorhexis was performed (sometimes under blue trypan). Lens material was aspirated with a Simcoe canula. After the bag was filled with the viscoelastic substance, a foldable lens was then injected gently in the posterior chamber. For all patients under 8 years old, a primary posterior capsulotomy was not routinely performed. An anterior vitrectomy was not possible in our setup. The viscoelastic substance was then removed from the anterior chamber with a Simcoe cannula. At the end of the procedure, a mixture of triancinolone and gentamicine was injected subconjunctival. Each patient started oral prednisolone (1 mg/kg/day) 3 days prior to the surgery and continued the medication for 7 days in the postoperative period.

Follow-up visits were scheduled as follows: 1 day, 7 days, 2 weeks, 12 weeks, 24 weeks and 52 weeks. At the first week follow-up visit, patients were examined on slit lamp to exclude any early complications. At the end of the second week, patients were submitted to cycloplegic refraction to assess the BCVA. All patient who did not improved at least two logMAR lines in BCVA in the absence of any surgical complication, were suspected of having amblyopia. They were referred for adequate management. In later visits, the posterior capsule status and other late complications were assessed. Patients who developed a posterior capsule opacification (PCO) were scheduled for a surgical posterior capsulotomy.

### Statistical analysis

Quantitative variables did not deviate significantly from the normal distribution; thus their values were reported as mean±standard deviation (SD). As results all tests carried out were parametric. Qualitative variables were presented in percent (%). Student’s paired t-test was carried out to compare post-operative and pre-operative visual acuity (VA). The analysis was performed using statistical software Epi-Info 7, IBM-SPSS 21, and R version 3.2.3. Since some expected frequencies were less than 5, Fisher’s exact test was used as measure of association. The Cochran–Armitage test for trend was used for ordinal categories. Odd ratios with 95% confidence interval (OR95%CI) were used for quantifying the associations. P-values less than 0.05 were considered statistically significant.

## Results

A total of 62 consecutive children from 5 to 15 years old underwent cataract surgery at the Mana Eye Clinic Nkongsamba between 2007 and 2015 under local anaesthesia. There were 33 males (53.22%) and 29 females (46.78%). The mean age at surgery was 10.18 ± 3.21. Our patients mostly originated from the Littoral region (43.58%). The details of patient demographic and clinical characteristics are summarised in Table 1. Nystagmus was present in 10 eyes (16.12%) at surgery. These 10 cases were classified as congenital cataract. Developmental cataract accounted for 37 cases (59.67%). Eye trauma was responsible for 17 cataracts (27.41%).

**Table 1:** Baseline demographic and clinical characteristics of patients included in this study

Characteristics	
**Gender N (%)**	
Male	33 (53.23)
Female	29 (46.77)
**Region of residency N (%)**	
Center	3(4.84)
Littoral	27 (43.54)
North-West	5 (8.06)
South-West	5 (8.06)
West	20 (32.26)
Non Cameroonian (Gabon)	2(3.22)
**Surgery**	
**Technics N (%)**	
MPA	40 (64.52)
SICS	22 (35.48)
IOL inplantation**N (%)**	54 (87.10)
Aphakia**N (%)**	8 (12.90)
Preoperative BCVA, log MAR mean ±SD (range)	1.19 ± 0.93 (0.6-2.3)
Postoperative BCVA, log MAR mean ±SD (range)	0.58 ± 0.88 (0.5-1.8)
follow up length (weeks) mean ±SD (range)	6.75 ± 3.36 (2- 13)
IOL implanted, dioptrymean ±SD (range)	22.01 ±3.169 (15- 29)
**Intraoperative Complications N (%)**	
Posterior capsule hole	4(6.45)
Lens sub luxation	3(4.80)
Iris dialyze and vitreous hemorrhage	1(1.61)
**Postoperative Complications N (%)**	
Posterior capsule fibrosis	16 (25.80)
Posterior synechia	6 (9.67)
Retinal detachment	2 (3.22)

**Figure 1:**
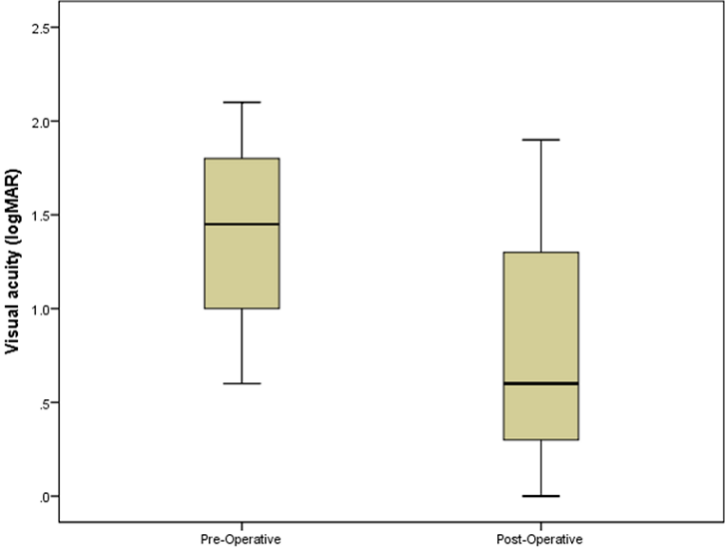
Comparison of the distributions of Visual acuity (logMAR) pre and post-operative

Biometry was done in 47 patients (75.80%). After undercorrection of 10%, the mean implanted IOL power was 22.01 ± 3.169. MPA was performed in 40 eyes (64.51 %), and 22 eyes (35.48%) underwent SICS. In 54 eyes, a posterior chamber IOL (48 in the capsule bag and 6 in the ciliar sulcus) was implanted (87.09%). Eight eyes (12.91%) were left aphakic. Primary posterior capsulotomy was performed in 33 eyes (53.22%). Preoperatively, all patients were blind in the affected eye. The mean BCVA (logMAR) increased significantly from 1.19 ± 0.93 preoperative to 0.58 ± 0.88 post-operative (p=0.000) (Figure 1). The postoperative changes in BCVA 2 weeks after the surgery are summarized in Table 2. Pairwise comparisons using Bonferroni-correction showed that visual outcome was better in developmental cataract group than congenital cataract group (p=0.000), and also than traumatic cataract group (p=0.002). Overall the visual outcome of congenital cataract was poor compared to traumatic and developmental cataract (p=0.000). Patients with development cataract were more likely to have higher change in their BCVA. Intraoperative complications included four posterior capsule holes with vitreous loss, three lens subluxations leading to vitreous loss and one case of iris dialysis with moderate intravitreal bleeding. The most common visually threatening late complications were posterior capsular opacity (16 patients, 25.80%), posterior synechia (6 patients, 9.67%) and retinal detachment (2 patients, 3.22%). The mean follow up length was 6.75 ± 3.36 b weeks (ranged 2- 13). Three months after surgery only 23 patients (37.09%) were seen. Of them 17 (73.91%) were from Littoral region. This visit was motivated by the non improvement in BCVA (8 patients), decrease in BCVA ( 9 patients) and need for second eye operation (6 patients).

**Table 2:** Changes in BCVA 2 weeks post-operative

Variation in logMAR lines	Congenital cataract n(%)	Developmental cataract n(%)	Traumatic cataract n(%)	Total n(%)	OR(95%CI)	P-value
[0,1]	6 (60.0)	1(2.86)	3(17.65)	10(16.13)	1	
[1-2 ]	4(40.0)	5(14.28)	5(29.41)	14(22.58)	5.0(0.39-138.34)	0.15
[2-3 ]	0(0.0)	7(20.0)	4(23.53)	11(17.74)	15.75(1.11-484.17)	0.01
4 and above	0(0.0)	22(62.86)	5(29.41)	27(43.55)	39.6(3.47-1060.72)	0.00001
**Total**	10(0.0)	35(100)	17(100.00)	62(100.0)		

## Discussion

Cataract is the leading cause of childhood blindness in Africa [6]. Early surgery is the only solution to restore sight and to prevent amblyopia. General anaesthesia provides good conditions for ocular surgery in children. This requires a well-equipped and experienced anaesthetic team [7]. In the current study, patients aged 5-15 years were operated under local anaesthesia. The use of peribulbar anaesthesia in paediatric cataract surgery has been reported by other authors [3, 8]. This is a viable option when facilities for safe and optimal general anaesthesia are unavailable. In our series, the mean age at surgery was 10.18 ± 3.21 years. This is higher than that reported by authors who used general anaesthesia for paediatric cataract surgery [9, 10]. The lack of general anaesthesia facilities appears to be one of the reasons for late surgery in our hospital. Other reported barriers for paediatric cataract surgery included: fear of the surgery, ignorance, economic problems and distance to the eye care facilities [9]. Biometry was done in 47 patients (75.80%). Although all patients were old enough to cooperate, keratometry was not possible for a significant number of participants during the surgery because of a machine defect. Many surgical techniques have been recommended for paediatric cataracts in the literature. Primary posterior capsulectomy and anterior vitrectomy are recommended for patients aged 8 years old or less [7]. Fourty patients in this study underwent MPA. This technique is more practical in children because the lenses are soft. It also incites less post operative inflammation and requires no suture. However, the cost and the availability of the foldable IOL constitute the major problems for this procedure in our environment. Technically, continuous circular capsulorhexis is the more difficult intraoperative step in young patients because of the anterior capsule structure. Usually, the anterior capsule extends toward the periphery. This “runaway rhexis” was the cause of a posterior capsule rise and vitreous loss in four cases (6.45%) in our series. Because of the lack of infrastructure, none of our patients underwent anterior vitrectomy as strongly recommended in the literature [11, 12]. This can explain why some patients developed PCO despite a primary capsulotomy. IOL implantation during paediatric cataract surgeries in the developing world is mandatory because other methods of visual rehabilitation (i.e., aphakic glasses and contact lenses) are less suitable in these settings. Primary posterior chamber IOL implantation in children has been shown to provide good visual outcomes [13].

In our series, primary IOL implantation was performed in 54 eyes (87.09%). The mean VA increased significantly from logMAR1.19 ± 0.33 preoperative to logMAR0.58 ± 0.88 postoperative (p = 0.000). Nystagmus was present in 16.12 % of patients before the surgery. It is a predictive factor for poor postoperative VA [5]. Children with congenital cataracts should ideally be operated on before the development of these factors, which occur in many cases after 10 weeks of age [14]. Delay of cataract surgery in children in our setting is linked to the absence of general anaesthesia facilities. Visual outcome was better in developmental cataract group than congenital cataract group (p=0.000), and also than traumatic cataract group (p=0.002). Severe visual deprivation was suspected to be the cause of poor changes in the postoperative BCVA in congenital cataract group while associated eye co morbidities affected the visual outcome in traumatic cataract group. Only 23 patients (37.09%) were seen 3 months after surgery. A similar situation had been reported in other developing countries where many patients are lost from follow up after surgery [15]. Reasons for withdrawal include distance to the hospital, financial difficulties, poor road infrastructure, ignorance and lack of awareness to the necessity of long-term follow-up care. Central posterior capsule opacification was the major late complication causing poor vision. Intact posterior capsule and absence of anterior vitrectomy favoured the PCO development [16]. We believe that primary anterior vitrectomy could have reduced the incidence of PCO and the risk of retinal detachment in our series.

The retrospective character constitutes a serious limitation to our study. Necessary information was not recorded in some files; therefore, the classification to congenital cataract was only based on clinical findings, such as nystagmus. We could not comment on the visual outcome of children suspected for amblyopia due to the fact that postoperative management of amblyopia was carried out in another hospital. The final visual outcome of some patients could have been affected because they were given a standard IOL.

## Conclusion

Our results suggest that pediatric cataract surgery can be done under local anaesthesia on selected patients presenting developmental cataract. In cases of congenital cataract, early surgical intervention and prompt optical rehabilitation is mandatory to prevent irreversible deprivational amblyopia. Creation of well-equipped and well-staffed centres for the treatment of paediatric cataracts should be a priority for decision makers in the developing world.

### What is known about this topic

- Pediatric cataracts are a common cause of childhood blindness;
- Pediatric cataract surgery must be done under general anesthesia.

### What this study adds

- Our study reports the use of local anesthesia in pediatric cataract surgery;
- It also highlights the need of well staffed and well equipped centers for pediatric surgery in developing countries;
